# Diagnostic performance of axillary ultrasound and standard breast MRI for differentiation between limited and advanced axillary nodal disease in clinically node-positive breast cancer patients

**DOI:** 10.1038/s41598-019-54017-0

**Published:** 2019-11-25

**Authors:** S. Samiei, T. J. A. van Nijnatten, H. C. van Beek, M. P. J. Polak, A. J. G. Maaskant-Braat, E. M. Heuts, S. M. J. van Kuijk, R. J. Schipper, M. B. I. Lobbes, M. L. Smidt

**Affiliations:** 1 Department of Surgery, Maastricht University Medical Centre+, Maastricht, The Netherlands; 2 Department of Radiology and Nuclear Medicine, Maastricht University Medical Centre+, Maastricht, The Netherlands; 3 GROW – School for Oncology and Developmental Biology, Maastricht University Medical Centre+, Maastricht, The Netherlands; 4 Department of Radiology, Maxima Medical Centre, Eindhoven, The Netherlands; 5 Department of Surgery, Maxima Medical Centre, Eindhoven, The Netherlands; 6 Department of Clinical Epidemiology and Medical Technology Assessment, Maastricht University Medical Centre+, Maastricht, The Netherlands

**Keywords:** Outcomes research, Surgical oncology

## Abstract

Preoperative differentiation between limited (pN1; 1–3 axillary metastases) and advanced (pN2–3; ≥4 axillary metastases) nodal disease can provide relevant information regarding surgical planning and guiding adjuvant radiation therapy. The aim was to evaluate the diagnostic performance of preoperative axillary ultrasound (US) and breast MRI for differentiation between pN1 and pN2–3 in clinically node-positive breast cancer. A total of 49 patients were included with axillary metastasis confirmed by US-guided tissue sampling. All had undergone breast MRI between 2008–2014 and subsequent axillary lymph node dissection. Unenhanced T2-weighted MRI exams were reviewed by two radiologists independently. Each lymph node on the MRI exams was scored using a confidence scale (0–4) and compared with histopathology. Diagnostic performance parameters were calculated for differentiation between pN1 and pN2–3. Interobserver agreement was determined using Cohen’s kappa coefficient. At final histopathology, 67.3% (33/49) and 32.7% (16/49) of patients were pN1 and pN2–3, respectively. Breast MRI was comparable to US in terms of accuracy (MRI reader 1 vs US, 71.4% vs 69.4%, p = 0.99; MRI reader 2 vs US, 73.5% vs 69.4%, p = 0.77). In the case of 1–3 suspicious lymph nodes, pN2–3 was observed in 30.4% on US (positive predictive value (PPV) 69.6%) and in 22.2–24.3% on MRI (PPV 75.7–77.8%). In the case of ≥4 suspicious lymph nodes, pN1 was observed in 33.3% on US (negative predictive value (NPV) 66.7%) and in 38.5–41.7% on MRI (NPV 58.3–61.5%). Interobserver agreement was considered good (k = 0.73). In clinically node-positive patients, the diagnostic performance of axillary US and breast MRI is comparable and limited for accurate differentiation between pN1 and pN2–3. Therefore, there seems no added clinical value of preoperative breast MRI regarding nodal staging in patients with positive axillary US.

## Introduction

The axillary lymph node status in breast cancer patients provides relevant information regarding treatment planning and prognosis^1,2^. In our current breast cancer workup, a preoperative axillary ultrasound (US) is routinely performed for evaluation of the axillary lymph nodes^3–5^. If suspicious axillary lymph nodes are detected with US, fine needle aspiration cytology (FNAC) or core needle biopsy is performed to identify clinically node-positive patients. Formerly, an axillary lymph node dissection (ALND) was performed in these clinically node-positive patients as standard practice. With increasing use of neoadjuvant systemic therapy (NST) and therefore the advantage of possible downstaging of the axilla, alternative approaches are proposed for the axillary lymph node staging, such as sentinel lymph node biopsy (SLNB), marking axillary lymph nodes with radioactive iodine seed (MARI), targeted axillary dissection (TAD), and combining radioactive iodine seed localisation in the axilla with the sentinel node procedure (RISAS), all to avoid ALND associated comorbidities^6–9^. By using such alternative approaches, the true pathological axillary lymph node status is unknown since these techniques have false-negative findings of up to 15%^6,7,10^. Combining an imaging technique before NST with these less invasive surgical staging methods, such as SLNB, MARI, TAD, or RISAS, can contribute to the selection of patients for whom further axillary treatment is (un)necessary.

In clinically node-negative patients treated with primary surgery, the role of a complementary ALND in sentinel node-positive breast cancer patients has been addressed in previous trials, such as the ACOSOG Z0011 and AMAROS^11,12^. These trials showed that omitting ALND or in the case of treatment with axillary radiotherapy in sentinel node-positive patients did not result in worse locoregional recurrence or survival rate^11–13^. When omitting (complementary) ALND or in the case of treatment with axillary radiotherapy, some patients with advanced axillary nodal disease (pN2–3; i.e., ≥4 axillary lymph node metastases) might not be identified. Patients with advanced axillary nodal disease are recommended to undergo radiation therapy of the chest wall or (reconstructed) breast with the addition of infraclavicular/supraclavicular lymph nodes, an imaging technique to identify pN2–3 disease before surgery is necessary.

Consequently, preoperative differentiation between limited (pN1; i.e., 1–3 axillary lymph node metastases) and advanced axillary nodal disease in breast cancer patients can provide relevant information regarding the surgical planning and guiding adjuvant radiation therapy. The role of breast MRI for evaluating axillary lymph node metastases has been described in earlier studies with reported sensitivity and specificity between 33.3–97.0% and 14.0–98.5%, respectively^14–16^. Hyun *et al*. showed that a negative breast MRI can exclude pN2–3 with negative predictive value (NPV) of 99.6%. However, the differentiation between pN1 and pN2–3 has not been addressed earlier in clinically node-positive patients and appears to be of clinical relevance.

Therefore, this study aimed to evaluate the diagnostic performance of preoperative axillary US and standard breast MRI for differentiation between pN1 and pN2–3 in clinically node-positive breast cancer patients. Furthermore, the second aim of this study was to evaluate whether an additional preoperative standard breast MRI is of added clinical value in patients with 1–3 suspicious axillary lymph nodes on US for differentiation between pN1 and pN2–3.

## Material and Methods

### Study population

All patients diagnosed with invasive breast cancer between January 2008 and December 2014, who had undergone preoperative axillary US and standard breast MRI, were retrospectively identified from two medical centres (Maxima Medical Centre (MMC) and Maastricht University Medical Centre+ (MUMC+)). Patients with preoperative axillary lymph node metastasis, confirmed by US-guided FNAC or core needle biopsy (i.e., clinically node-positive patients), were included for analyses. All patients had undergone primary breast surgery and ALND. Data on the patient, tumour, and treatment characteristics were retrospectively collected. The medical ethics committee of MMC and MUMC + approved this study and waived the necessity to acquire informed consent due to the retrospective study design.

### Ultrasound evaluation

The preoperative axillary lymph node status was determined by the axillary US performed by dedicated breast radiologists. Different US systems were used for the axillary US examinations: Aplio XG (Toshiba Medical Systems Europe, Zoetermeer, the Netherlands) with a linear 12 MHz array transducer, ATL-HDI5000 (Philips Healthcare, Best, the Netherlands) with a linear 5–12 MHz array transducer, and iU22-xMATRIX (Philips Healthcare, Best, the Netherlands) with a linear 2–17 MHz array transducer. During the axillary US examination, the patient was positioned with the ipsilateral hand placed behind the head if possible. The axillary region was examined in a standardised approach, starting at the low axilla (level I: inferior and lateral to the pectoralis minor muscle) and continuing upwards toward mid-axilla (level II: medial and lateral to the pectoralis minor muscle and interpectoral). The apical axilla (level III: superior and medial to the pectoralis minor muscle with apical lymph nodes) was only evaluated when suspicious axillary lymph nodes were found in level I and/or level II. In the case of suspicious axillary lymph node(s), FNAC or a 16–18 gauge core needle biopsy was performed of the most suspicious lymph node. The total number of suspicious lymph nodes was estimated and reported. Characteristics of suspicious axillary lymph nodes on US included round or irregular shape, cortical thickness greater than 2.3 mm, diffuse cortical thickening, effacement or replacement of fatty hilum, and increased peripheral blood flow^17–21^.

### Breast MRI acquisition and analysis

All included patients had undergone a standard breast MRI in the prone position using a 1.5 Tesla MRI scanner (Philips Healthcare, Best, the Netherlands). In MMC, they started using the Philips Gyroscan NT scanner in 2008 with a body coil which was later replaced with an 8-channel breast coil. In 2013, the MRI scanner was replaced by Philips Ingenia R4.2 scanner with a 16-channel breast coil. In MUMC+, two types of MRI scanners, Philips Ingenia and Intera, were used with a body coil that was replaced with a 16-channel breast coil in 2011. Overview of MR protocols can be found as Supplementary Material A online.

**Figure 1:**
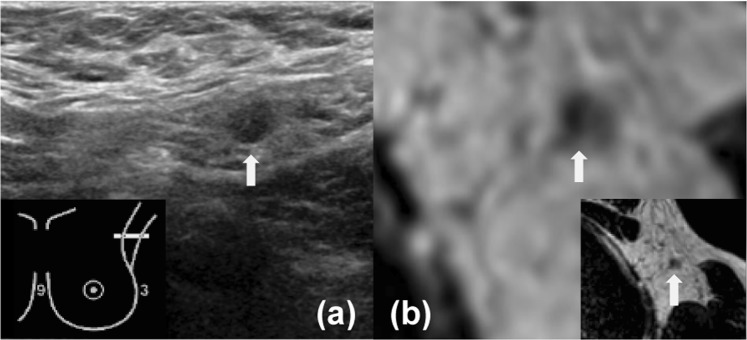
Images of the axilla of a 52-year-old female patient with a 34 mm large invasive ductal carcinoma in her left breast, which was treated with mastectomy and ALND. For both US and MRI (reader 1 and 2) N1 axillary lymph node disease was reported. The white arrow indicates the suspicious lymph node on US and MRI. Histopathology of the ALND reported pN2–3 (largest diameter, 14 mm). (**a**) Axillary US (**b**) Transversal unenhanced T2-weighted breast MRI.

**Figure 2:**
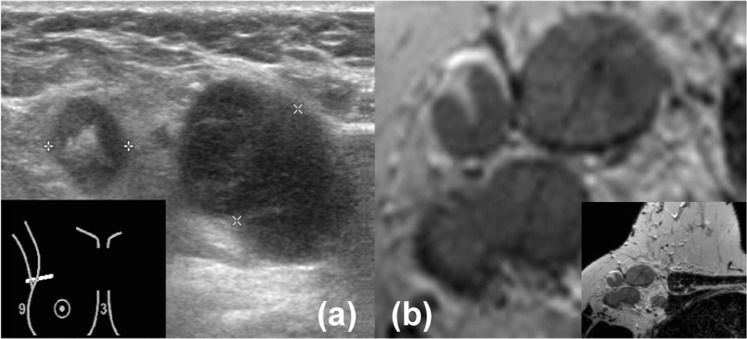
Images of the axilla of a 55-year-old female patient with a 31 mm large invasive lobular carcinoma in her right breast, which was treated with mastectomy and ALND. For both US and MRI (reader 1 and 2) N2–3 axillary lymph node disease was reported. Histopathology of the ALND reported pN2–3 (largest diameter, 50 mm). (**a**) Axillary US (**b**) Transversal unenhanced T2-weighted breast MRI.

The breast MRI exams were pre-screened by a radiology resident (T.J.A.v.N.) with three years of breast imaging experience. Screening of the MRI exams for eligibility was based on the complete field of view (FOV) of the axillary region, the absence of movement artifacts, and adequate signal-to-noise ratio. Two breast radiologists with 7 years (H.C.v.B. [reader 1]) and 14 years (M.P.J.P. [reader 2]) of experience with breast MRI retrospectively evaluated all axillary lymph nodes (i.e., axillary level 1–3) on the unenhanced T2-weighted MR sequence. Each lymph node on the MRI exams was scored using a confidence scale (0, no axillary lymph nodes; 1, definitely benign; 2, probably benign; 3, probably malignant; 4, definitely malignant) (Figs. 1 and 2)^22^. Characteristics of suspicious axillary lymph nodes on MRI included irregular margins, inhomogeneous cortex, perifocal oedema, asymmetry, and absence of fatty hilum or chemical shift artifact^22,23^. These criteria were used to classify an axillary lymph node as either negative or positive. Similar to clinical practice, the radiologists were aware of the tumour side, clinical tumour size as assessed on MRI, and the clinical axillary lymph node status. However, they were blinded from each other’s results and had no information about the final pathological axillary lymph node status.

### Clinical and pathological axillary lymph node status

The clinical nodal status was based on the total number of suspicious axillary lymph nodes (N1: 1–3 suspicious lymph nodes; N2–3: ≥4 suspicious lymph nodes). The ALND lymph nodes (i.e., pathological axillary lymph nodes) were paraffin-embedded after formalin fixation for histological assessment. All harvested lymph nodes with a diameter larger than 5 mm were sliced with a maximum thickness of 3 mm. All slides were stained with hematoxylin and eosin. Isolated tumour cells (≤0.2 mm and/or <200 cells in a single histological cross-section) and micrometastases (0.2 ≤ 2.0 mm) were considered as negative, and macrometastases (>2.0 mm) as positive axillary lymph nodes. The number of axillary lymph node metastases on final histopathology defined the pathological axillary lymph node status. Pathological N1 disease was defined by 1–3 axillary lymph node metastases and pathological N2–3 disease was defined by ≥4 axillary lymph node metastases^24^.

### Statistical analysis

The confidence scale was dichotomised into benign (lymph nodes scores 0–2) and malignant (lymph nodes scores 3–4). Histopathology served as the gold standard. Diagnostic performance parameters (sensitivity, specificity, positive predictive value (PPV), NPV, accuracy) were calculated for axillary US and breast MRI for differentiation between pN1 and pN2–3. Sensitivity and specificity were defined as probabilities that in the case of pN1 or pN2–3, imaging showed 1–3 or ≥4 suspicious axillary lymph nodes, respectively. PPV and NPV were defined as probabilities that in the case of 1–3 and ≥4 suspicious axillary lymph nodes on imaging, the patient truly had pN1 and pN2–3, respectively. Comparison of sensitivity, specificity, and accuracy of US and MRI were performed with the McNemar test for paired proportions. The PPV and NPV of US and MRI were compared by using the generalised score test statistics^25^. In the subgroup analysis, the diagnostic performance parameters of breast MRI for differentiation between pN1 and pN2–3 were calculated for patients who had 1–3 suspicious axillary lymph nodes on US. The diagnostic performance parameters were presented with 95% confidence intervals (CIs). The reported CIs and p-values were two-sided and 0.05 was used as a cutoff for significance. Interobserver agreement between the two radiologists for evaluating the breast MRI exams was calculated by using Cohen’s kappa coefficient (k < 0.2 = poor, 0.21–0.40 = fair, 0.41–0.60 = moderate, 0.61–0.80 = good, 0.81–1.0 = very good)^26^. Statistical analyses were performed by using R project software (version 3.5.1, R Foundation for Statistical Computing, Vienna, Austria).

## Results

A total of 49 patients [mean age, 57 years; range, 34–79 years] were included for analyses; 17 patients from MMC and 32 patients from MUMC + . Mastectomy was performed in 41 (83.7%) patients and breast-conserving surgery was performed in 8 (16.3%) patients. The most common tumour histology and receptor subtype were of ductal origin (81.6%) and ER + HER2- subtype (67.3%), respectively. At final histopathology, 67.3% (33/49) and 32.7% (16/49) of the patients had pN1 and pN2–3 axillary lymph node disease, respectively. The mean size of the macrometastases was 15 mm (range, 6–50 mm). Patient, tumour, and treatment characteristics are summarised in Table 1.

**Table 1:** Patient, tumour, and treatment characteristics.

	All patients (n = 49)
Age (years) (mean; range)	57 [34–79]
Clinical tumour size (mm) (mean; range)	35 [4–100]
**Clinical tumour stage (%)**	
T1	9 (18.4)
T2	31 (63.2)
T3	9 (18.4)
**Breast surgery (%)**	
Breast-conserving surgery	8 (16.3)
Mastectomy	41 (83.7)
**Pathological nodal stage (%)**	
pN1	33 (67.3)
pN2	9 (18.4)
pN3	7 (14.3)
**Tumour histology (%)**	
Ductal	40 (81.6)
Lobular	8 (16.3)
Mixed ductal and lobular	1 (2.1)
**Uni - or multifocal tumour (%)**	
Unifocal	36 (73.5)
Multifocal and/or multicentric	13 (26.5)
**Tumour grade (%)**	
1	4 (8.2)
2	26 (53.1)
3	19 (38.7)
**Receptor status (%)**	
ER + HER2+	3 (6.1)
ER + HER2−	33 (67.4)
ER-HER2 +	6 (12.2)
Triple negative	7 (14.3)

Abbreviations: ER, Estrogen Receptor; HER2, Human Epidermal growth factor Receptor 2; Triple negative, negative for ER, PR, and HER2.

### Diagnostic performance of axillary ultrasound

Of the 49 patients classified as clinically node-positive on US, 46 (93.9%) were classified as having N1 axillary lymph node disease and 3 (6.1%) were classified as having N2–3 axillary lymph node disease. In the case of 1–3 suspicious axillary lymph nodes evaluated on US, pN2–3 was found in 30.4% of the patients with a PPV of 69.6%. In the case of ≥4 suspicious axillary lymph nodes evaluated on US, pN1 was found in 33.3% of the patients with an NPV of 66.7% (Table 2).

**Table 2:** Diagnostic performance of axillary US and standard breast MRI for differentiation between pN1 and pN2–3.

	US (n = 49)	MRI reader 1 (n = 49)	p-value^a^ US vs MRI1	MRI reader 2 (n = 49)	p-value^b^ US vs MRI2
Sensitivity	97.0% (32/33) [84.2–99.9]	84.8% (28/33) [68.1–94.9]	0.10	84.8% (28/33) [68.1–94.9]	0.10
Specificity	12.5% (2/16) [1.6–38.3]	43.8% (7/16) [19.8–70.1]	0.03	50.0% (8/16) [24.7–75.3]	0.01
PPV	69.6% (32/46) [54.2–82.3]	75.7% (28/37) [58.8–88.2]	0.13	77.8% (28/36) [60.8–89.9]	0.06
NPV	66.7% (2/3) [9.4–99.2]	58.3% (7/12) [27.7–84.8]	0.77	61.5% (8/13) [31.6–86.1]	0.85
Accuracy	69.4% (34/49) [54.6–81.7]	71.4% (35/49) [56.7–83.4]	0.99	73.5% (36/49) [58.9–85.1]	0.77

Abbreviations: US, ultrasound; PPV, positive predictive value; NPV, negative predictive value; MRI1, MRI reader 1; MRI2, MRI reader 2.

^a^McNemar and generalised score test for comparison of diagnostic performance parameters between US and breast MRI of reader 1.

^b^McNemar and generalised score test for comparison of diagnostic performance parameters between US and breast MRI of reader 2.

Data in parenthesis are absolute numbers. Data in brackets are 95% confidence intervals.

### Diagnostic performance of standard breast MRI

Of the 49 patients that were clinically node-positive on US, reader 1 classified 37 (75.5%) patients as having N1 axillary lymph node disease and 12 (24.5%) patients as having N2–3 axillary lymph node disease on breast MRI. Reader 2 classified 36 (73.5%) patients as having N1 axillary lymph node disease and 13 (26.5%) patients as having N2–3 axillary lymph node disease on breast MRI. In the case of 1–3 suspicious axillary lymph nodes evaluated on breast MRI, pN2–3 was found in 22.2–24.3% of the patients with a PPV of 75.7–77.8%. In the case of ≥4 suspicious axillary lymph nodes evaluated on breast MRI, pN1 was found in 38.5–41.7% of the patients with an NPV of 58.3–61.5% (Table 2). Interobserver agreement between the two radiologists for reviewing the breast MRI exams was considered good (k = 0.73).

Breast MRI of reader 1 was comparable to US in terms of both sensitivity (84.8% vs 97.0%, p = 0.10), PPV (75.7% vs 69.6%, p = 0.13), NPV (58.3% vs 66.7%, p = 0.77), and accuracy (71.4% vs 69.4%, p = 0.99). Breast MRI of reader 1 showed better specificity (43.8% vs 12.5%, p = 0.03) compared to US. Breast MRI of reader 2 was comparable to US in terms of both sensitivity (84.8% vs 97.0%, p = 0.10), PPV (77.8% vs 69.6%, p = 0.06), NPV (61.5% vs 66.7%, p = 0.85), and accuracy (73.5% vs 69.4%, p = 0.77). Breast MRI of reader 2 also showed better specificity (50.0% vs 12.5%, p = 0.01) compared to US.

The subgroup analysis showed that 46 patients had N1 axillary lymph node disease on US. Reader 1 correctly classified 75.0% (27/36) of the patients as having pN1 axillary lymph node disease and 50.0% (5/10) of the patients as having pN2–3 axillary lymph node disease on breast MRI. Reader 2 correctly classified 77.1% (27/35) of the patients as having pN1 axillary lymph node disease and 54.5% (6/11) of the patients as having pN2–3 axillary lymph node disease on breast MRI (Table 3). Interobserver agreement between the two radiologists for reviewing the subgroup breast MRI exams was considered good (k = 0.69).

**Table 3:** Diagnostic performance of standard breast MRI for differentiation between pN1 and pN2–3 if ultrasound showed 1–3 suspicious axillary lymph nodes.

	MRI reader 1 (n = 46)	MRI reader 2 (n = 46)
Sensitivity	84.4% (27/32) [67.2–94.7]	84.4% (27/32) [67.2–94.7]
Specificity	35.7% (5/14) [12.8–64.9]	42.9% (6/14) [17.7–71.1]
PPV	75.0% (27/36) [57.8–87.9]	77.1% (27/35) [59.9–89.6]
NPV	50.0% (5/10) [18.7–81.3]	54.5% (6/11) [23.4–83.3]
Accuracy	69.6% (32/46) [54.2–82.3]	71.7% (33/46) [56.5–84.0]

Abbreviations: PPV, positive predictive value; NPV, negative predictive value.

Data in parenthesis are absolute numbers. Data in brackets are 95% confidence intervals.

## Discussion

Non-invasive preoperative differentiation between pN1 and pN2–3 has become increasingly important since this can provide relevant information regarding surgical planning and guiding adjuvant radiation therapy. In this study, we showed that the diagnostic performance of preoperative axillary US and standard breast MRI is comparable and inaccurate for differentiation between pN1 and pN2–3 in clinically node-positive breast cancer patients. Furthermore, when US showed 1–3 suspicious axillary lymph nodes, an additional preoperative standard breast MRI correctly diagnosed pN2–3 in only 50.0–54.5% of these patients.

ALND and radiation therapy to the chest wall or (reconstructed) breast and infraclavicular/supraclavicular lymph nodes are recommended in breast cancer patients with ≥4 axillary lymph node metastases who are at significant risk of locoregional recurrence in the chest wall or conserved breast and nodal basins, regardless of the response to NST^27–29^. The number of positive nodes is not only important for guiding adjuvant radiation therapy, but also the timing of breast reconstruction in patients treated with primary surgery. Previous research has demonstrated that radiation therapy on the reconstructed breast can increase complication rates and may compromise the aesthetic outcomes, especially in implant-based reconstructions^30–33^. Therefore, preoperative identification of pN2–3 disease is preferable in patients with a desire for immediate breast reconstruction to avoid potential radiation therapy complications.

In the diagnostic workup of breast cancer patients, the US and US-guided FNAC or core needle biopsy are widely accepted for the assessment of axillary lymph node metastases given the low morbidity, cost-effectiveness, and high accuracy^17,34^. Previous studies have addressed the ability of axillary US for differentiation between pN0-N1 and pN2-N3^17,35^. They reported that a negative axillary US can exclude pN2–3 with an NPV of 96.0–97.7%^17,35^. In the case of a positive axillary US, the ability to exclude pN2–3 was considered insufficient with a reported NPV of 58.5–71%^35,36^. This is in line with our results. We demonstrated that the ability to exclude pN2–3 was 69.6% in the case of 1–3 suspicious axillary lymph nodes on US.

A standard breast MRI is frequently added to the diagnostic workup of breast cancer patients for a more accurate display of tumour size or extent. Since breast MRI includes the complete FOV of the axillary region in 1 out of 3 patients, it may provide the radiologist with the opportunity to evaluate axillary lymph node metastases^36^. Compared to the US, MRI has the advantage to visualise deeper-lying lymph nodes, the ability to assess the contralateral axilla at the same time, and lack of operator dependence^37^. An additional breast MRI in clinically node-positive patients has not been studied before. Hyun *et al*. reported that by adding breast MRI to negative cases of US and US-guided biopsy, pN2–3 was excluded in 98.0% of the patients^38^. In our study, we added a breast MRI to positive cases of US-guided biopsy. We showed that an additional breast MRI in clinically node-positive patients excluded pN2–3 in 75.7–77.8% of the patients. However, 38.5–41.7% of the patients were incorrectly classified as N2–3 axillary lymph node disease on breast MRI.

Since US and breast MRI are of limited value in clinically node-positive patients for differentiation between pN1 and pN2–3, the focus should perhaps shift to other imaging modalities. Other promising non-invasive imaging modalities are being investigated for the assessment of axillary lymph node metastases, such as PET/CT and PET/MRI. In the meta-analysis of Liang *et al*., the diagnostic performance of MRI and PET/CT was compared for the assessment of axillary lymph node metastases^39^. They showed that MRI (94.0%) has a higher pooled area under the ROC curve compared to PET/CT (88.0%) for diagnosing axillary lymph node metastases^39^. With the introduction of hybrid PET/MRI systems, the diagnostic advantages of MRI and PET are combined. Melsaether *et al*. showed that PET/MRI improves the diagnostic performance of axillary lymph node metastases detection from 88.0% to 100% compared to PET/CT^40^. In the Nijnatten *et al*. study, dedicated axillary PET/MRI was compared to other imaging modalities (i.e., US, MRI, and PET/CT)^41^. PET/MRI resulted in a nodal status change of 40.0–75.0% compared to US and MRI, respectively, and a nodal status change of 22.0% compared to PET/CT^41^. However, not all studies on PET/CT and PET/MRI take into account the number of lymph node metastases.

In the present study, size and morphology were used for the assessment of axillary lymph node metastases on the unenhanced T2-weighted MR sequence. The unenhanced MR imaging seems to be one of the most promising sequences for axillary lymph node metastases assessment due to the anatomical characterisation of the axillary lymph nodes^42,43^. It has been previously shown that the use of a dedicated axilla coil improves the accuracy of axillary lymph node staging^44–47^. However, it was chosen to use the size and morphology on the unenhanced T2-weighted breast MR sequence to facilitate possible implementation in other hospitals.

This study has certain limitations. The analyses were based on a small group of patients. This might have caused clinically meaningful differences in diagnostic performance to go undetected. However, the differentiation between pN1 and pN2–3 has not been studied before in clinically node-positive patients. Sample size expansion is not possible given that nowadays almost every clinically node-positive patient receives NST. In addition, the diagnostic performance parameters were based on a patient-by-patient level instead of a node-by-node level. All patients were treated with ALND, but the precise correlation between the visualised suspicious lymph nodes on US and breast MRI and the pathological results were not available, resulting in no node-by-node analysis. Further, different MRI scanners with varying protocols were used in the two medical centres. Given the small sample size in this study, a comparison between MR protocols was not possible. However, all MRI systems and protocols conform to the guidelines of the European Society of Breast Imaging. Finally, in this study, a dedicated axillary MR protocol was not available. A dedicated axilla coil could have improved the diagnostic performance parameters of MRI. Although, all MRI exams were pre-screened for a complete FOV of the axillary region.

## Conclusion

In clinically node-positive breast cancer patients, the diagnostic performance of preoperative axillary US and standard breast MRI is comparable and both imaging modalities are unable to accurately differentiate between pN1 and pN2–3. An additional preoperative standard breast MRI can correctly diagnose pN2–3 in only 50.0–54.5% of the patients when US showed 1–3 suspicious axillary lymph nodes. Based on this study, there is no added clinical value of preoperative standard breast MRI for differentiation between pN1 and pN2–3 in patients with positive axillary US.

## Supplementary information

Supplementary Material A

## Data Availability

The datasets generated during and/or analysed during the current study are available from the corresponding author on reasonable request.
