# Supplementary material for: Diagnostic performance of axillary ultrasound and standard breast MRI for differentiation between limited and advanced axillary nodal disease in clinically node-positive breast cancer patients
Source: Sci Rep. 2019 Nov 25;9:17476. doi: 10.1038/s41598-019-54017-0 (PMC6877558; doi:10.1038/s41598-019-54017-0)
Supplement: Supplementary file 1 — Supplementary Material A [file 41598_2019_54017_MOESM1_ESM.pdf]

Diagnostic performance of axillary ultrasound and standard breast MRI for differentiation between limited and advanced axillary nodal disease in clinically node-positive breast cancer patients

S. Samiei, MD, T.J.A. van Nijnatten, MD, PhD, H.C. van Beek, MD, M.P.J. Polak, MD,
A.J.G. Maaskant-Braat, MD, PhD, E.M. Heuts, MD, PhD, S.M.J. van Kuijk, PhD,
R.J. Schipper, MD, PhD, M.B.I. Lobbes, MD, PhD*, M.L. Smidt, MD, PhD*

*shared last authorship

Supplementary Material A. Overview of MR protocols for standard breast MRI

Maxima Medical Centre, Eindhoven, The Netherlands

MRI 1: Philips Gyroscan NT 1.5 Tesla

	2008	2009	2010	2011	2012	2013
Coil	Sense body coil	Sense body coil	Sense body coil	Sense breast 4-channel/ Sense body coil	Sense breast 4-channel/ Sense body coil	Sense breast 4-channel/ Sense body coil
Pixel size (mm)	0.7 x 0.7	0.7 x 0.7	0.7 x 0.7	0.7 x 0.7	0.7 x 0.7	0.7 x 0.7
Repetition time (ms)	2500	2500	2500	2500	2500	2500
Echo time (ms)	100	100	100	100	100	100
Echo train length	24	24	24	24	24	24
Flip angle (°)	90	90	90	90	90	90
Slice thickness (mm)	2.5	2.5	2.5	2.5	2.5	2.5

MRI 2: Philips Ingenia R4.2

	2013	2014
Coil	Sense breast 16-channel	Sense breast 16-channel
Pixel size (mm)	0.8 x 0.8	0.8 x 0.8
Repetition time (ms)	3957	3957
Echo time (ms)	110	110
Echo train length	23	23
Flip angle (°)	90	90
Slice thickness (mm)	2.5	2.5

Maastricht University Medical Centre+, Maastricht, The Netherlands

MRI 1: Philips Intera 1.5 Tesla

	2009	2010	2011	2012	2013	2014
Coil	Sense body coil	Sense body coil	Sense breast 16-channel	Sense breast 16-channel	Sense breast 16-channel	Sense breast 16-channel
Pixel size (mm)	0.7 x 0.7	0.6 x 0.6	1.0 x 1.0	1.0 x 1.0	1.0 x 1.0	1.0 x 1.0
Repetition time (ms)	5922	3012	2000	2000	2000	2000
Echo time (ms)	110	110	204	205	205	258
Echo train length	33	33	80	80	80	96
Flip angle (°)	90	90	90	90	90	90
Slice thickness (mm)	3.0	3.0	2.0	2.0	2.0	2.0

MRI 2: Philips Ingenia 1.5 Tesla

	2009	2010	2011	2012	2013	2014
Coil	Sense body coil	Sense body coil	Sense breast 16-channel	Sense breast 16-channel	Sense breast 16-channel	Sense breast 16-channel
Pixel size (mm)	0.7 x 0.7	0.6 x 0.6	0.9 x 0.9	0.9 x 0.9	0.9 x 0.9	0.9 x 0.9
Repetition time (ms)	8213	6845	2000	2000	2000	2000
Echo time (ms)	110	110	215	218	218	218
Echo train length	29	29	95	95	95	95
Flip angle (°)	90	90	90	90	90	90
Slice thickness (mm)	3.0	3.0	2.0	2.0	2.0	2.0
